# A Lightweight YOLOv4-Based Forestry Pest Detection Method Using Coordinate Attention and Feature Fusion

**DOI:** 10.3390/e23121587

**Published:** 2021-11-27

**Authors:** Mingfeng Zha, Wenbin Qian, Wenlong Yi, Jing Hua

**Affiliations:** School of Software, Jiangxi Agricultural University, Nanchang 330045, China; feng_ai@outlook.com (M.Z.); yiwenlong@mail.ru (W.Y.); 15870668662@163.com (J.H.)

**Keywords:** pest detection, YOLOv4, MobileNet, attention mechanism, feature fusion, deep learning

## Abstract

Traditional pest detection methods are challenging to use in complex forestry environments due to their low accuracy and speed. To address this issue, this paper proposes the YOLOv4_MF model. The YOLOv4_MF model utilizes MobileNetv2 as the feature extraction block and replaces the traditional convolution with depth-wise separated convolution to reduce the model parameters. In addition, the coordinate attention mechanism was embedded in MobileNetv2 to enhance feature information. A symmetric structure consisting of a three-layer spatial pyramid pool is presented, and an improved feature fusion structure was designed to fuse the target information. For the loss function, focal loss was used instead of cross-entropy loss to enhance the network’s learning of small targets. The experimental results showed that the YOLOv4_MF model has 4.24% higher mAP, 4.37% higher precision, and 6.68% higher recall than the YOLOv4 model. The size of the proposed model was reduced to 1/6 of that of YOLOv4. Moreover, the proposed algorithm achieved 38.62% mAP with respect to some state-of-the-art algorithms on the COCO dataset.

## 1. Introduction

Forestry is crucial in national defense construction, industrial and agricultural production, daily life, and national economic construction [1]. By detecting pests quickly and accurately, the effectiveness of pest measurement and reporting can be guaranteed. However, pest detection mainly relies on expert systems, which involve a large and cumbersome workload. Much research on automatic pest detection has been proposed to improve the efficiency of pest detection. At present, automated pest detection methods can be divided into two main categories: sensor-based methods [2,3,4,5] and visual image-based methods [6,7,8,9,10,11,12,13,14,15,16].

Sensor-based methods are costly and ineffective, making it challenging to promote this technology. Therefore, many researchers are turning to visual imagery. Traditional vision methods mainly rely on the manual design of relevant features. While detection can achieve better results compared to that of sensor-based methods in specific fields, they are laborious, time-consuming, and not expandable. With the development of deep learning, automated feature extraction based on convolutional neural networks can extract rich information from images [17]. However, deep learning-based approaches face some challenges: (1) the detection of small targets is difficult; (2) models deployed in mobile or embedded devices pose difficulties in the balance of recognition effectiveness and light weight.

To solve the above problems, based on the You Only Look Once (YOLO) algorithm, a lightweight end-to-end pest detection algorithm, YOLOv4_MF, is proposed. The experimental results showed that the proposed algorithm achieved a 4.24% improvement in mAP and 39 FPS on the pest dataset compared to YOLOv4. In summary, the main contributions of this paper are as follows:

1. To increase the mobile detection speed of a deployed model, MobileNetv2 embedded with coordinate attention was used as the feature extraction network, and deep separable convolution was applied instead of ordinary convolution.
2. A three-layer symmetric Spatial Pyramid Pooling (SPP) network was constructed to integrate the information of different types of feature maps. Not only the diversity of the images was improved and the convergence of the model was accelerated, but also the overfitting of the network was prevented.
3. To integrate the semantic and detailed information of small targets and introduce as few parameters as possible, the BA block was designed as the multi-scale feature fusion network.
4. The distribution of positive and negative samples in the dataset was uneven, and difficult samples were present. For these, focal loss was used for the classification, and confidence loss to make the model more accurate in recognizing small targets.

## 2. Related Work

In this section, research on vision-based pest detection is reviewed. The approaches are divided into traditional methods and deep learning methods.

Among researchers describing the traditional object detection methods, Xie et al. [18] used sparse coded histograms to quantify original features such as the color and shape of insects and further used multicore learning to fuse multiple features. Deng et al. [19] proposed to detect regions of interest in images using saliency maps, combined multiple schemes to enrich image information and extract it, and finally a support vector machine for classification. Qin et al. [20] proposed to define the logarithmic spectrum of an image as the novel part of image information and to transform the remaining spectrum to the null domain to obtain edge detection. Xie et al. [21] used dictionary coding to obtain the underlying features of images and then a multilevel classifier to classify the pests. Yang et al. [22] used an SVM-based approach to identify insects with different wing sizes.

Object detection algorithms based on deep learning use convolutional neural network instead of the traditional manual feature selection. They can be divided into two categories. One class includes two-stage object detection models represented in the region with CNN features series [23,24,25], which have high accuracy in terms of object localization and detection rate. Still, their real-time performance needs to be improved. The other class includes one-stage detection models represented by YOLO [26,27,28] and the single-shot multibox detection series [29,30,31], which are fast in detection but less accurate compared with the two-stage models and are less effective in detecting small targets. With the two-stage detection models, object detection is considered a classification problem. The detection results are obtained by first generating regions containing objects and then classifying and calibrating the candidate regions. In contrast, the one-stage detection models treat the object detection problem as a regression problem and supply the final detection results.

Deep learning algorithms have better generalization ability and higher robustness than traditional algorithms [32]. Currently, for small targets such as forestry pests, the detection ability is unsatisfactory for three main reasons. First, less information can be extracted. Minor objects occupy fewer pixels in the image and carry less information, making it challenging to extract discriminative features without being affected by surrounding environmental factors. Second, the requirements for positioning accuracy are high. Whether in the training or in the prediction process, the offset of the bounding box is large for the error of small target detection. Third, the object aggregation problem exists. When it occurs, after being presented to the deep feature map through multiple downsampling, targets will be clustered into one point, resulting in the inability to distinguish different objects. Besides, it will make the bounding boxes difficult to regress, and the model difficult to converge.

This paper proposes a detection algorithm for small targets such as forestry pests, which ensures the lightweight of the model and improves detection.

## 3. YOLOv4 Network Model

**Figure 1:**
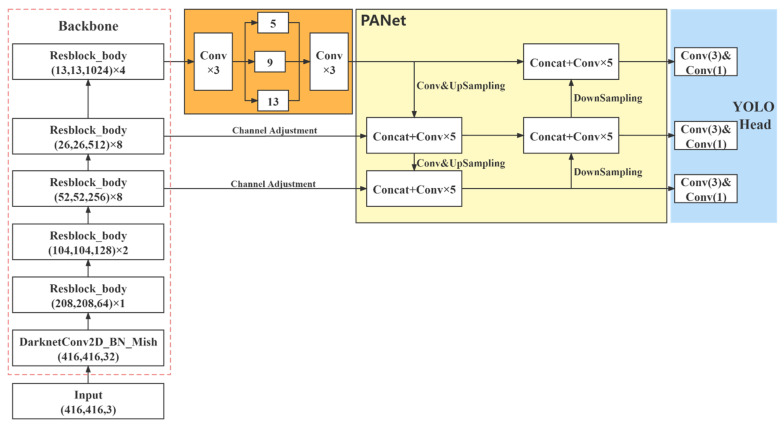
YOLOv4 network structure, including the feature extraction network, the SPP structure, the feature fusion, and the prediction network.

The model of YOLOv4 [33] is composed of three parts, i.e., the backbone feature extraction network (backbone), the feature pyramid (neck), and the prediction end (head). The network structure when the resolution of the input image is 416×416 pixels is shown in Figure 1.

In the backbone, CSPDarkNet53 is used to extract features. The neck is composed of the SPP network and the PANet network for feature fusion. The extracted features are transformed using multiple convolutions in the head to obtain the prediction results.

**Figure 2:**
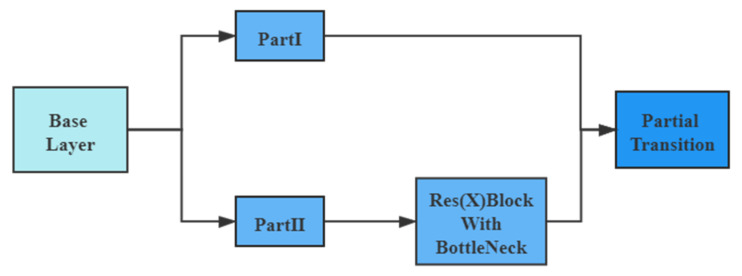
Resblock_body structure, consisting of a Res(X) Block and a residual edge.

CSPDarkNet53 was developed based on Darknet53, drawing on the experience of CSPNet [34] and improving the activation function. When the image is imported into the network, the three channels of the original image are adjusted to 32 channels. Then, feature extraction is applied through Resblock_body blocks, whose repetitions are 1, 2, 8, 8, 4. Resblock_body prevents the duplication of gradient information in network optimization, thus reducing the computational effort of the inference process. The Resblock_body structure is shown in Figure 2.

The feature mapping of the base layer is first divided into two parts and then merged across the stage hierarchy. The Res(X) block is made by stacking one downsampling and multiple residual structures.

**Figure 3:**
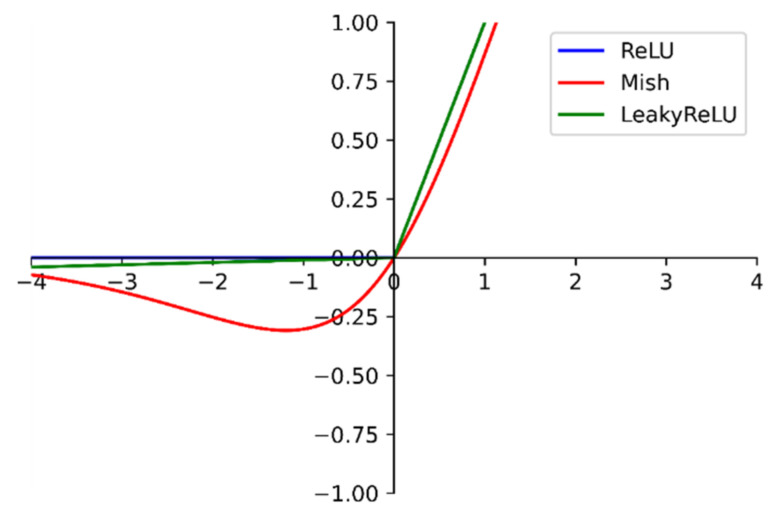
Comparison of the Mish function with each activation function. The Mish function curve is smoother.

The activation function from the LeakyReLU function in Darknet53 is modified to the Mish [35]. The Mish function is shown in Figure 3.

At the negative level, unlike the zero bound of the ReLU [36] function and the linear transformation of the LeakyReLU function, the Mish function is a section of a smooth curve. At the same time, it also behaves more smoothly at the positive level. The smoothness allows the information to penetrate the network better, resulting in better accuracy and generalization [35]. Therefore, this study used the Mish function to replace the ReLU6 function of the inverse residual structure in MobileNetv2.

**Figure 4:**
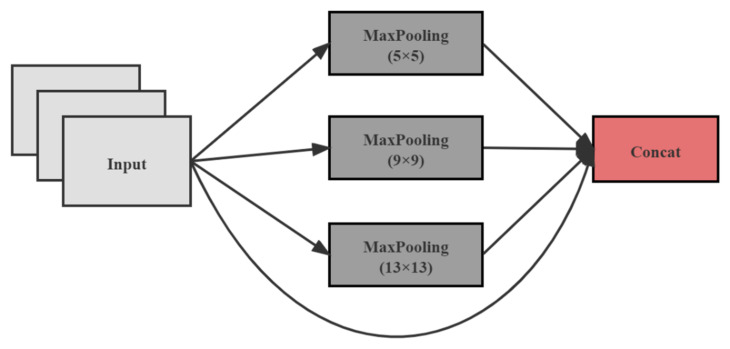
SPP structure, consisting of four different maxpoolings.

Before feature fusion, the last class of feature maps was subjected to the SPP [37] structure. The SPP structure is shown in Figure 4. 

The SPP structure, using maxpooling kernels such as {1×1, 5×5, 9×9, 13×13}, stitches feature maps of different scales. Compared with simply using the k×k maxpooling, it can more effectively increase the receiving range of backbone features and significantly separate the most critical context features. 

In YOLOv3, a top-down feature pyramid network (FPN) is used as the feature fusion structure of the network to transfer high-level semantic information to the lower layers. However, YOLOv4 adds a bottom-up feature pyramid containing two PAN structures after the FPN layer to form a PANet feature fusion structure. Semantic features conveyed from top to bottom by the FPN and positional features conveyed from bottom to top by the feature pyramid merge each other. The parameters of different detection layers are aggregated from different trunk layers.

YOLOv4 extracts the last three feature maps in the shapes of (52,52,256), (26,256,512), and (13,13,1024), respectively. Depending on the dataset, the final output of the network varies. Let us take the COCO dataset as an example, which contains 80 categories. Since there are three prior frames for each feature layer and each initial frame contains four location information and one confidence information, the final output channel dimension is 3×(80+5)=255.

In the prediction stage, the prediction frames are selected using DIOU_NMS to obtain the best one. In addition, YOLOv4 has also improved loss function and training techniques.

## 4. Proposed Approach: YOLOv4_MF

### 4.1. Backbone: MobileNetv2

The MobileNet [38] network was proposed by the Google team in 2017, focusing on lightweight networks in mobile or embedded devices. Compared with the traditional convolutional neural network, it dramatically reduces the model parameters and operations with a slight reduction in accuracy. The MobileNet network reduces the model parameters to 1/32 of VGG16, with 0.9% accuracy loss. 

Compared with other network models, the main reason for the significant reduction in parameters and computation of the MobileNet model is the use of deep separable convolution instead of ordinary convolution. The ratio between the depth-wise separable convolution computation and the ordinary convolution computation is:

(1) $$\begin{array}{l} \frac{D_{K}\cdot D_{K}\cdot M\cdot D_{F}+M\cdot N\cdot D_{F}\cdot D_{F}}{D_{K}\cdot D_{K}\cdot M\cdot N\cdot D_{F}\cdot D_{F}}=\frac{1}{N}+\frac{1}{D_{K}^{2}} \end{array}$$

where $D_{F}$ denotes the height and width of the input feature map, and M denotes the number of channels. $D_{K}$ denotes the height and width of the convolution kernel, and N denotes the number of channels of the output feature map.

When the convolution kernel size is 3, the ratio is $\frac{1}{N}+\frac{1}{9}$. Theoretically, the ordinary convolution is about 9 times more computationally intensive than the deep separable convolution.

**Figure 5:**
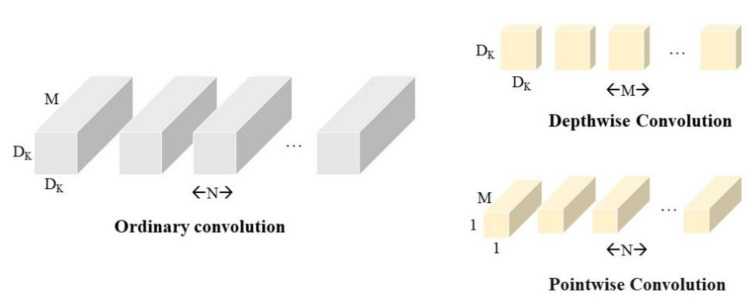
Comparison of ordinary convolution and deep divisible convolution. The latter consists of Depth-wise and Pointwise convolution.

The depth-wise separable convolution structure is shown in Figure 5.

In the traditional convolution process, the channels of the input feature map and the channels of the convolution kernel are equal, and the channels of the output feature map and the convolution kernels are equal. In contrast, the number of channels of the convolution kernel is 1, and that of the channels of the input feature map and the channels of the output feature map is equal to the number of convolution kernels.

**Figure 6:**
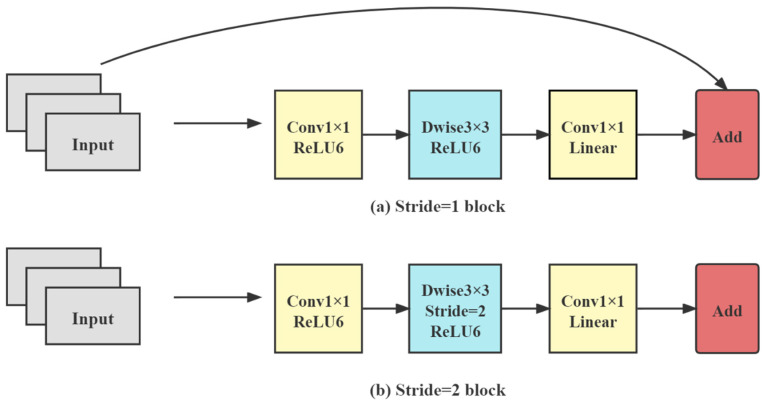
Inverse residual structure. Depending on whether the kernel stride is 1, it can be divided into two categories.

In MobileNetv2 [39], the core part is the inverse residual structure, as shown in Figure 6.

Each inverse residual network consists of two 1×1 ordinary convolutions and a 3×3 depth-wise separated convolution. First, the network expands the dimensionality of feature maps by 1×1 convolution, then extracts features by 3×3 depth-wise separated convolution, and finally compresses the channels by 1×1 convolution. For activation functions, on the one hand, the ReLU6 activation function can make the model more robust when using low-precision calculations. On the other hand, it can increase nonlinearity in high-dimensional space but is damaged in lower dimensions. The linear activation function performs better in low-dimensional space. Therefore, the two ordinary convolutions are activated by the ReLU6 and Linear functions, respectively, and ReLU6 activates the depth-wise separated convolution.

Depending on the step size and on whether the input and output feature maps have the same shape, the inverse residual structure can be divided into two categories. Let us add a shortcut when the step size is 1, and the input feature matrix and the output feature matrix have the same shape. Otherwise, no shortcut is used. In the overall network structure, blocks with step size 1 are used first, and blocks with step size 2 are used in the middle layer.

An expansion factor is set in the network, allowing the network to accommodate different application requirements. Table 1 shows the changes in the input and output of the feature map after adding the expansion factor t. In this paper, the value of t was 6.

### 4.2. Attention Mechanism: Coordinate Attention

Coordinate attention [40] is a lightweight and efficient attention mechanism that embeds location information into channel attention, allowing mobile networks to acquire knowledge over a larger area. Learning from the experimental procedure of coordinate attention, this paper introduced the attention mechanism in the inverted residual structure of MobileNetv2.

**Figure 7:**
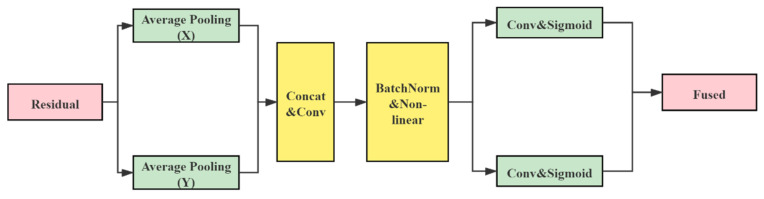
Coordinate attention structure, including coordinate information embedding and attention generation.

Coordinate attention encodes remote dependencies and location information from horizontal and vertical spatial directions and then aggregates the features. The structure diagram is shown in Figure 7; it includes two steps: coordinate information embedding and attention generation.

#### 4.2.1. Coordinate Information Embedding

The characteristics of global pooling determine makes it challenging to retain location information, so the pooling needs to be decomposed to capture location information spatially. Specifically, let us decompose the pooling along with both horizontal and vertical directions. The outputs of the c-th channel with height h and width w are expressed respectively as:

(2) $$z_{c}^{h}(h)=\frac{1}{W}\sum_{0\leq i<W}x_{c}(h,i)$$

(3) $$z_{c}^{w}(w)=\frac{1}{H}\sum_{0\leq j<H}x_{c}(j,w)$$

where H and W denote the height and width of the pooling kernel.

The above two transformations aggregate features along with two spatial directions. They generate a pair of direction-aware feature maps that enable the module to capture dependencies along one spatial path and retain accurate location information along the other [40].

#### 4.2.2. Attention Generation

Let us splice the above two transformations in spatial dimension and use 1×1 convolution to compress the channels. BatchNorm and Non-linear are then used to encode the spatial information in the vertical and horizontal directions. Let us segment the encoded information and adjust the channels of the attention map to equal the number of channels of the input feature map using 1×1 convolution. Then, let us use the sigmoid function for normalization and weighted fusion. The final output can be expressed as follows

(4) $$y_{c}(i,j)=x_{c}(i,j)\cdot g_{c}^{h}(i)\cdot g_{c}^{w}(j)$$

where $x_{c}(i,j)$ denotes the input feature map, and $g_{c}^{h}(i)$, $g_{c}^{w}(i)$ denotes the attention weights of the two spatial directions.

### 4.3. Multi-Scale Feature Fusion: BA Block

As the network layers deepen, the semantics of the features change from low-dimensional to high-dimensional. However, each layer of the network causes some degree of feature loss. High-level features are rich in semantic information for object classification, while low-level features are rich in fine-grained information for object localization. Therefore, an efficient feature fusion structure was constructed to integrate the advantages of high and low levels.

**Figure 8:**
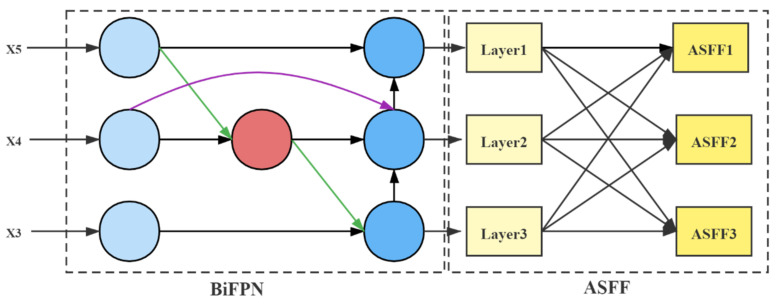
BA block structure, consisting of two parts, BiFPN and ASFF.

The BA module consists of the Weighted Bi-directional Feature Pyramid Network (BiFPN) [41] and the Adaptive Spatial Feature Fusion (ASFF) [42]. By introducing few parameters, the detection effect was improved. Following the initial cross-scale weighted feature fusion by BiFPN, the features were imported to ASFF for a more profound integration of pest information. Figure 8 illustrates the structure of the BA Block.

#### 4.3.1. Composition of the BiFPN

BiFPN has two features. One feature is cross-scale connectivity, which obtains information from different resolution feature maps; the other one is weighted fusion, which assigns corresponding weights to the importance of varying input features. The weighting calculation process is as follows

(5) $$M=\sum_{i}\frac{w_{i}}{\varepsilon +\sum_{j}w_{{}_{j}}}\times I_{i}$$

In Equation (5), let us use the ReLU function to activate $w_{i}$ so that $w_{i}\geq 0$, and ε is 0.0001 to avoid the denominator being 0. $I_{i}$ represents the value of the i input feature.

Given a set of multi-scale features $X=(X_{1}^{in},X_{2}^{in},\ldots )$, $X_{i}^{in}$ denotes the features of layer i. The feature fusion network uses the input features of levels 3, 4, and 5 (obtained by downsampling the original image by 8×, 16×, and 32×, respectively). Taking the input image of 416×416 as an example, $X_{3}^{in}$ denotes the feature layer with a resolution of 52×52. BiFPN can iterate with a basic unit iteratively. For visualization, the process of fusing the upper and lower layers is described by mathematical derivation in one iteration:

(6) $$X_{4}^{td}=Conv(\frac{w_{1}\cdot X_{4}^{in}+w_{2}\cdot Deconv(X_{5}^{in})}{w_{1}+w_{2}+\varepsilon })$$

(7) $$X_{4}^{out}=Conv(\frac{w_{1}^{\prime }\cdot X_{4}^{in}+w_{2}^{\prime }\cdot X_{4}^{td}+w_{3}^{\prime }\cdot Resize(X_{3}^{out})}{w_{1}^{\prime }+w_{2}^{\prime }+w_{3}^{\prime }+\varepsilon })$$

(8) $$X_{4}^{out}=Swish(X_{4}^{out})$$

where $X_{4}^{td}$ denotes the intermediate features of the sixth layer, $X_{4}^{out}$ denotes the output features of the sixth layer, $X_{5}^{out}$ matches the resolution with $X_{4}$ layer by deconvolution, and $X_{4}^{out}$ is activated by the Swish [43] activation function after fusion. 

#### 4.3.2. Composition of ASFF

Although BiFPN performs the initial feature fusion, more information fusion is required for small targets. Simply repeating iterations of BiFPN base units cannot achieve detection accuracy and lightweight balance. Therefore, this paper introduces the ASFF network that executes weighted fusion by setting self-learning weights for each fused feature map. This method is superior to direct concatenation, additive, or fast normalized fusion [42]. We used $X_{3}^{out}$, $X_{4}^{out}$ and $X_{5}^{out}$ as inputs to ASFF. The procedure and principles of ASFF are as follows.

(a) Feature Adjustment

ASFF-3, ASFF-2, ASFF-1 correspond to $X_{3}^{out}$, $X_{4}^{out}$, and $X_{5}^{out}$, respectively. To get ASFF-3, we adjusted the channels of $X_{4}^{out}$ and $X_{5}^{out}$ to the same as $X_{3}^{out}$ by 1×1 convolution and then adjusted them to the same width and height by upsampling (interpolation). Similarly, to obtain ASFF-1, it was necessary to downsample $X_{3}^{out}$ and $X_{4}^{out}$ using a convolution of size 3×3 with a step size of 2. For $X_{4}^{out}$, it was also necessary to use a maximum pooling with a step size of 2.

(b) Adaptive Fusion

The weight parameters α, β, γ were obtained by adjusting the shape of the feature map after 1×1 convolution, and satisfy the formula α+β+γ=1 (α, β, γ values are located in [0, 1]). The adjusted feature maps were multiplied by the corresponding weights to obtain the new fusion features at the corresponding levels. The weights were calculated as follows:

(9) $$\alpha_{ij}^{l}=\frac{e^{\alpha_{\alpha_{ij}}^{l}}}{e^{\alpha_{\alpha_{ij}}^{l}}+e^{\beta_{\alpha_{ij}}^{l}}+e^{\gamma_{\alpha_{ij}}^{l}}}$$

where $\alpha_{\alpha_{ij}}^{l}$, $\beta_{\alpha_{ij}}^{l}$, and $\gamma_{\alpha_{ij}}^{l}$ are the control parameters of the three weights, respectively; $\alpha_{ij}^{l}$ corresponds to the weights of l at three different levels at the (i,j) position.

The new ASFF layer was calculated as follows:

(10) $$W_{ij}^{l}=\alpha_{ij}^{l}\times A_{ij}^{1\to l}+\beta_{ij}^{l}\times A_{ij}^{2\to l}+\gamma_{ij}^{l}\times A_{ij}^{3\to l}$$

where $A_{ij}^{n\to l}$ denotes the feature vector located at (i,j) after adjusting the feature map $A_{ij}^{n}$ to the same size as $\alpha_{ij}^{l}$, $\beta_{ij}^{l}$, $\gamma_{ij}^{l}$ and has the same meaning as $\alpha_{ij}^{l}$.

### 4.4. Loss Function

The loss function of YOLOv4 consists of the bounding box regression loss, confidence, and classification loss. Previously, the most frequently used bounding box regression loss was IoU_Loss. The mathematical expression of IoU is:

(11) $$IoU=\frac{\left|A\cap B\right|}{\left|A\cup B\right|}$$

In Equation (11), A and B represent the prediction and target frames, respectively.

However, there are two problems. When the prediction frame and the target frame do not intersect, Equation (11) cannot reflect the distance between the two structures. At this time, the function is not derivable and cannot be optimized. The second issue is that the IoU is the same, but the positions of the prediction frames are different, so IoU_Loss cannot distinguish the difference between the intersection of the two. Zheng et al. [44] proposed DIoU_loss to solve the above problems by considering the overlapping area and the distance of the center point of the two frames. CIoU_Loss [44] introduces the aspect ratio of the two frames based on DIoU_Loss v, making it possible to converge faster and obtain better regression results when the intersection ratio is 0. The expressions for the length/width ratio v and CIoU_Loss, $L_{CIoU}$, are as follows:

(12) $$v=\frac{4}{\pi^{2}}(\arctan\frac{w^{gt}}{h^{gt}}-\arctan\frac{w^{p}}{h^{p}})$$

where $\frac{w^{gt}}{h^{gt}}$ denotes the aspect ratio of the target frame, and $\frac{w^{p}}{h^{p}}$ denotes the aspect ratio of the predicted frame;

(13) $$L_{CIoU}=1-IoU+\frac{\rho^{2}(b,b^{gt})}{c^{2}}+\alpha v$$

b and $b^{gt}$ represent the prediction frame’s center points and the target frame, respectively; ρ represents the Euclidean distance between the two center points, and c represents the diagonal length of the smallest closed area containing the target frame’s prediction frame at the same time.

To address the positive and negative sample imbalance, focal loss [45] is used to replace the cross-entropy loss as the confidence and classification loss of the network. It assigns a higher loss weight to the foreground images, so that the model focuses more on the classification of the foreground.

(14) $$L_{FL}=\left\{\begin{array}{r} -\alpha {\left(1-p\right)}^{\gamma }\log(p),y=1\\ -(1-\alpha )p^{\gamma }\log(1-p),y=0 \end{array}\right.$$

In this paper, α was 1, and γ was 2.

In the end, the loss of the model can be expressed as $L_{Total}$, as shown in Equation (15).

(15) $$L_{Total}=L_{CIoU}+2\times L_{FL}$$

### 4.5. YOLOv4_MF Network Model

**Figure 9:**
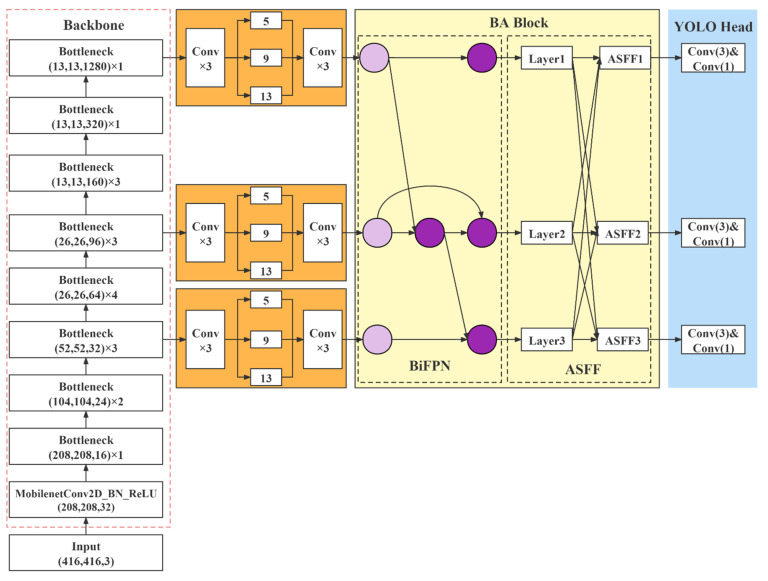
YOLOv4_MF network structure, consisting of MobileNetv2, SPP network, BA network, and prediction network.

The proposed YOLOv4_MF network structure is shown in Figure 9.

YOLOv4_MF used MobileNetv2 with fused coordinate attention as the feature extraction network to obtain feature maps with shapes (52,52,32), (26,26,96), (13,13,1280). Then, the feature maps were extracted by the SPP structure for different levels of features. After that, the BA feature fusion network integrated the input information. Finally, the network head parsed the feature map to obtain the detection results.

## 5. Experimental Results and Analysis

### 5.1. Dataset, Environment, and Parameters

The target detection dataset in this paper included 2183 images of forestry pests produced by Beijing Forestry University. The data format was JPEG, and the pest species were divided into seven categories: Boerner, Leconte, Linnacus, acuminatus, armandi, coleoptera, and linnaeus. The training set contained 1693 images, the validation set included 245 images, and the remaining images were used as the test set. Since there were no linnaeus data in the validation set, this paper chose the remaining six classes from the original training set as the new training set. The format of the dataset was generated by referring to the VOC dataset. Table 2 shows the statistical information about the dataset.

Online data enhancement was performed by mixup [46], random level flipping, random cropping, random affine transformation, etc.

The pest target dataset was trained based on the pre-trained model to accelerate network convergence. The hardware environment and software versions for the experiments are shown in Table 3.

The key parameters of the experiment were set as shown in Table 4.

**Figure 10:**
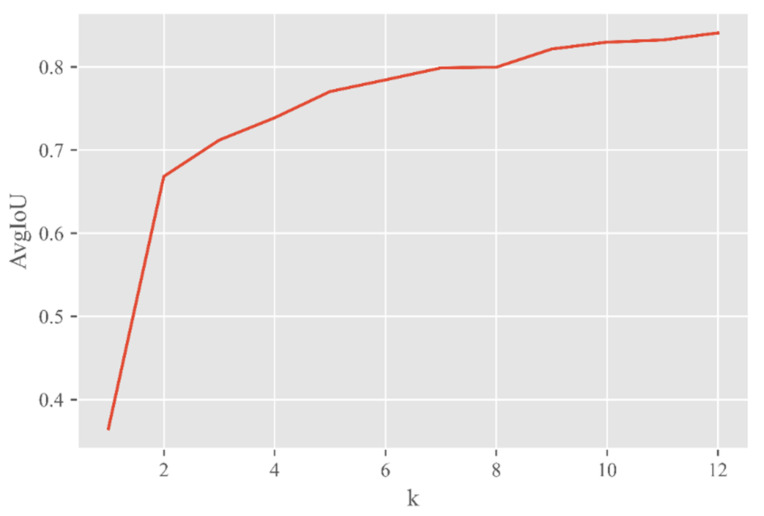
The change curve of AvgIoU as k varies from 1 to 12.

Most of the target frames in the pest dataset occupied less than 2% of the whole image, so the network needed to pay more attention to targets of small sizes. However, the anchor frames preset by the YOLOv4 model were based on the COCO dataset and were not suitable for small target detection. Therefore, this paper used the K-means algorithm for clustering analysis. With the clustering center k varying from 1 to 12, the average intersection over union (AvgIoU) was used as a measure to select the appropriate k value and reset the size of the candidate box. The variation curve shown in Figure 10 was obtained.

With the increasing value of k, AvgIou tended to be stable. When k was greater than 9, the AvgIoU increased less and less. Therefore, the value of k was 9 in this paper. The sizes of the nine candidate frames were (13,20), (17,29), (19,13), (22,28), (27,17), (28,45), (29,23), (41,42), (45,28).

**Figure 11:**
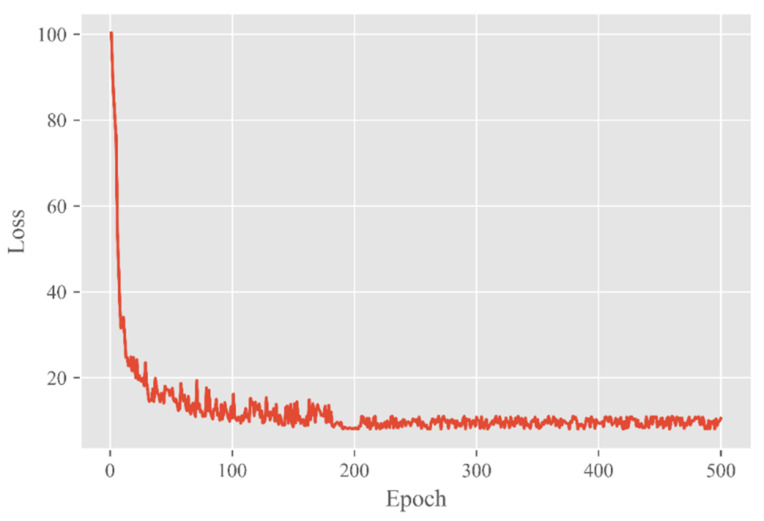
The change curve of loss as epoch varies from 1 to 500.

To facilitate the volatility of the network training, the loss values of the first few rounds are not shown in Figure 11.

It can be seen from the figure that the loss values kept decreasing as a general trend until convergence. When the number of epochs was 200, the loss curve had no apparent oscillation, and the loss value was stable at about 10. 

**Figure 12:**
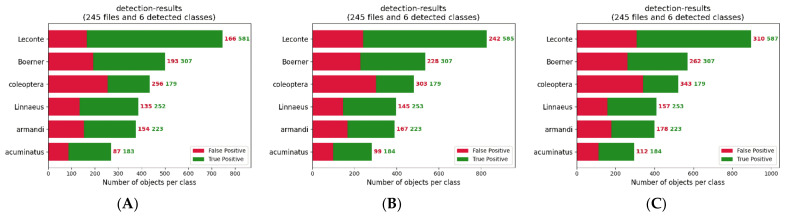
(**A**–**C**) results of different categories of detection for IoU = 0.3, 0.45, and 0.5, respectively.

In object detection, when the IoU value between the predicted frames and the actual frames is higher than a certain threshold, it is considered that the model outputs the correct results. If the value of IoU is set too high, it will cause the loss of some right prediction results; if the value of IoU is set too low, it will cause some wrong prediction results to be unfiltered. Experiments were conducted at different IoU values to obtain the results shown in Figure 12.

The setting of the IoU value also affects the precision and recall of pest identification. If the IoU is set too high, the accuracy will decrease. Accordingly, the accuracy rate will increase if IoU is set too low, but the recall rate will decrease. So IoU = 0.5 was chosen as a standard.

### 5.2. Evaluation

To verify that the improved YOLOv4 model has superior performance, it is possible to measure mAP, FPS, size, etc.

Let us obtain AP values for each category by calculating the area enclosed by the accuracy and recall curves (P–R curves) with the coordinate axes. Then, the AP values of six classes are averaged to obtain the mAP. Some performance metrics are defined as follows:

(16) $$Precision=\frac{TP}{TP+FP}$$

(17) $$Recall=\frac{TP}{TP+FN}$$

(18) $$F1\text{-}score=2\cdot \frac{Precision\cdot Recall}{Precision+Recall}$$

(19) $$mAP=\frac{1}{N}\sum_{i=1}^{N}\int_{0}^{1}PrecisiondRecall$$

N represents the number of detected categories, TP means that the actual class of the sample is positive, and the model predicts positive cases; FN implies that the correct classification of the example is positive, but predicts negative points; FP indicates that the proper category of the sample is negative, but the prediction is positive.

### 5.3. Performance Comparison

Table 5 shows the results of comparing YOLOv4_MF with outstanding models under different metrics.

The FPS of YOLOv4_MF is six points higher than that of YOLOv4; YOLOv4_MF is not much different from YOLOv4_tiny and SSD_MobileNet in terms of detection speed, model operations, and the number of parameters. The Linnaeus and acuminatus class AP values of YOLOv4_MF improved by 7.42% and 3.42% over those of YOLOv4, 39.34% and 16.43% over those of YOLOv4_tiny, and 14.87% and 13.38% over those of SSD_MobileNet. Optimal results are also achieved in terms of accuracy and recall. In terms of mAP, YOLOv4_MF improved by 4.24%, 17.26%, and 17.67% with respect to YOLOv4, YOLOv4_tiny, and SSD_MobileNet, respectively.

**Figure 13:**
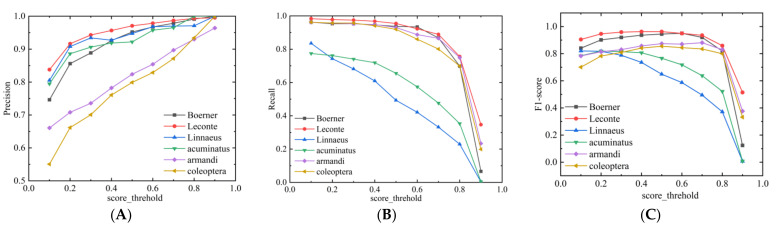
(**A**–**C**) Change curves of precision, recall, and F1-score, respectively.

In Figure 13, when the score_threshold was 0.5, the precision for five categories was above 80% (for the remaining class, it was close to 80%) and for four of them, it was even higher than 90%. It was found that YOLOv4_MF is effective in improving the precision. Four categories had a recall greater than 90%, and the remaining two categories havd a lower recall. However, the overall effect was still good.

The above data, on the one hand, demonstrate the effectiveness of the new feature extraction network and depth-wise separable convolution in model lightweighting. On the other hand, they also reflect the effect of attention mechanism, symmetric SPP structure, and BA structure on detection accuracy and miss detection rate.

**Figure 14:**
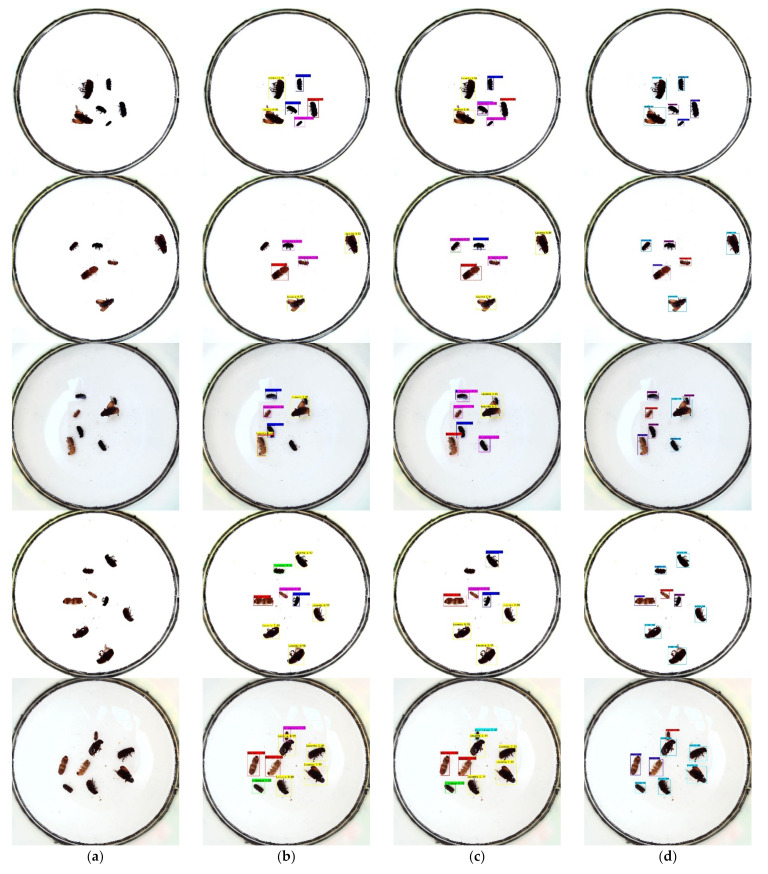
Comparison diagram of the actual detection of different algorithms. (**a**) Original diagram; (**b**) YOLOv4_tiny detection diagram; (**c**) YOLOv4 detection diagram; (**d**) YOLOv4_MF detection diagram.

In Figure 14, the first column shows the original image, the second column shows the detection result of YOLOv4_tiny, the third column shows the detection result of YOLOv4, and the fourth column shows the detection result of YOLOv4_MF.

In the first row, YOLOv4_tiny showed a significant deviation in the detection frame and did not accurately frame the target, while YOLOv4 had some duplicate frames. In the second row, YOLOv4_tiny missed detecting the Linnaeus object. In the third row, YOLOv4_tiny missed two targets and misdetected one target, while YOLOv4 missed a tiny target near the larger target. YOLOv4 missed one target in the fourth row. All three models in the fifth row performed better. It can be seen that the YOLOv4_MF algorithm has more accurate prediction frame localization and higher accuracy and recall in target detection.

To verify that the proposed algorithm has good mobility, we also conducted experiments on the MS COCO dataset. Table 6 shows the results of the YOLOv4_MF algorithm compared with some mainstream algorithms.

When the IoU thresholds were set to 0.5 and 0.5:0.95, the mAP values of YOLOv4_MF improved by 7.89%, 0.87% with respect to the two-stage Faster-RCNN, by 15.58%, 8.06% with respect to the one-stage RetinaNet algorithm, and by 11.96%, 1.84% with respect to the anchor-free CenterNet algorithm. When IoU was 0.5, the mAP value was 0.56% and 27.78% higher than that of YOLOv3 and YOLOv4_tiny, respectively.

It was found that the YOLOv4_MF algorithm achieved the best experimental results with a smaller input size, which demonstrates that the YOLOv4_MF algorithm has good detection performance and relocatability.

## 6. Conclusions

To detect forestry pests efficiently and quickly, the YOLOv4_MF deep neural network is proposed. By integrating BiFPN and ASFF, a BA module was constructed to obtain effective features. It was applied to three feature maps generated by MobileNetv2, with embedded coordinate attention and SPP network for feature fusion. To solve the sample imbalance problem during training and optimize the model parameters, focal loss was introduced. In addition, the 3×3 ordinary convolution was replaced by the deep separable convolution to further reduce the model parameters. The Mish activation function was used in the backbone network to facilitate the transfer of feature information. Compared with YOLOv4, YOLOv4_tiny, and SSD_MobileNet, YOLOv4_MF had the highest mAP value (88.93%) in the pest dataset. Furthermore, the size of the model was optimized compared to that of the base model for deployment in mobile devices in the field. The study was effective in improving the detection of small targets. It makes a contribution to the deployment of deep learning models in embedded and mobile devices for pest identification counting and other applications. However, false detection problems remain. In the future, we will conduct further research to improve the acquisition of target contextual information.

## Figures and Tables

**Table 1:** MobileNetv2 Expansion Factor t.

Input	Transform	Output
h×w×k	1×1 Conv, ReLU6	h×w×(tk)
h×w×(tk)	3×3 DW, ReLU6	$\frac{h}{s}\times \frac{w}{s}\times (tk)$
$\frac{h}{s}\times \frac{w}{s}\times (tk)$	1×1 Conv, Linear	$\frac{h}{s}\times \frac{w}{s}\times k^{\prime }$

**Table 2:** Target Statistics of the Pest Dataset.

Categories	Number of Goals	
	Training Set	Validation Set
Boerner	1595	318
Leconte	2216	594
coleoptera	2091	186
armandi	1765	231
Linnaeus	818	292
acuminatus	953	235
Total	9438	1856

**Table 3:** Experimental Environment Configuration.

Hardware and Software	Configuration Parameter	
Computer	Operating System:	Ubuntu18.04
	CPU:	Intel (R) Xeon (R) CPU E5-2678 v3 @ 2. 50 GHz
	GPU:	NVIDIA Tesla K80
	RAM:	8 GB
	Video memory:	12 GB
Software	Python3.7 + PyTorch1.8.1 + CUDA11.1 + cuDNN8.0.5 + Opencv4.5.2 +Pycharm2019.3.1	

**Table 4:** Experimental Parameter Settings.

Parameter	Value
Bacth_size	4
Cumulative times before optimization	4
Learning rate	1 × 10^−3^
Warm-up epochs	5
Number of iterations	500
Image size	416 × 416
Optimizer	Adam

**Table 5:** Comparison Results for Different Indicators.

		YOLOv4	YOLOv4_tiny	SSD_MobileNet	YOLOv4_MF
FPS		33	45	42	39
Total FLOPs (GFLOPs)		30.17	3.43	3.77	4.05
Params Size (MB)		245.53	22.74	27.68	38.10
AP	Boerner	95.74%	88.10%	80.30%	**95.75%**
	Leconte	94.28%	93.21%	82.82%	**98.13%**
	coleoptera	83.81%	73.57%	67.05%	**90.60%**
	armandi	87.06%	72.82%	67.52%	**91.03%**
	Linnaeus	74.73%	42.81%	67.28%	**82.15%**
	acuminatus	72.54%	59.53%	62.58%	**75.96%**
Precision		86.02%	73.85%	78.38%	**90.39%**
Recall		74.73%	51.44%	31.44%	**81.41%**
F1-score		0.79	0.57	0.42	**0.84**
mAP(%)		84.69%	71.67%	71.26%	**88.93%**

**Table 6:** Performance Comparison of Different Algorithms on the COCO Dataset.

Type	Input Size	mAP@0.50	mAP@0.50:0.95
RetinaNet	1333 × 1333	53.22%	32.25%
CenterNet	512 × 512	56.84%	38.47%
Faster-RCNN	-	60.91%	39.44%
YOLOv3	416 × 416	67.22%	38.12%
YOLOv4_tiny	416 × 416	41.02%	21.59%
**YOLOv4_MF**	416 × 416	67.78%	38.62%

## Data Availability

Not applicable.
